# Hybrid FFF/CNC: An open source hardware & software system

**DOI:** 10.1016/j.ohx.2024.e00536

**Published:** 2024-05-17

**Authors:** Luis Vincent Tejada Martinez, Jean-François Witz, Denis Najjar, Xavier Boidin, François Lesaffre, Vincent Martin, Sophie Badin, Emmanuel Berte

**Affiliations:** CNRS: UMR 9013 - LaMcube - Laboratoire de Mécanique, Multiphysique, Multiéchelle, Lille, France; Centrale Lille, Cité Scientifique, 59650 Villeneuve-d’Ascq, France; Polytech Lille, Av. Paul Langevin, 59655 Villeneuve-d’Ascq, France

**Keywords:** Hybrid, FFF, CNC, Additive manufacturing, Subtractive manufacturing, Milling

## Abstract

This paper presents a low-cost milling system composed of spindle mountable on a multi tool 3D printer equipped with maxwell kinematic coupling (E3D “ToolChanger” in this article) as well as two open-source software solutions for implementing a hybrid FFF/CNC manufacturing process. The first solution is the use of a traditional CAM software (FreeCad) for machining programming through the development of a dedicated post-processor. The second is an automatic layer-by-layer hybridization enabled by the software “SuperSlicer”. This method requires no machining knowledge but only allows contouring operations. Results of experiments show that the spindle presented in this work is capable of successfully carrying out a hybrid process that significantly improves the surface roughness parameters, with an improvement factor of 10 for most parameters. An uniformization of surface roughness parameters was also observed in the construction direction and in the deposition/machining direction. The layer-by-layer hybridization yields the better results in terms of surface roughness. This is because the reduced depth of cut (equivalent to a printed layer) minimizes stress and temperature rise, resulting in highly favorable cutting conditions.

## Graphical abstract

**Figure:**
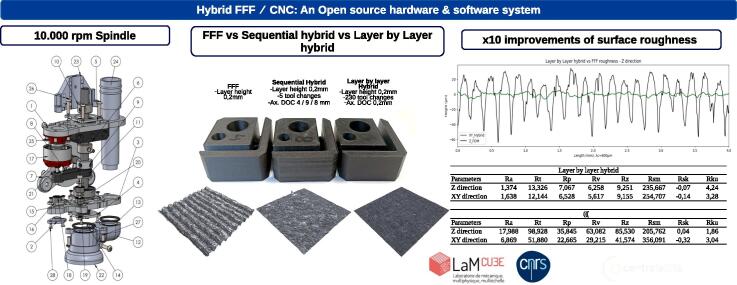

Specifications table

**Table:** 

Hardware name	Hybrid FFF/CNC: An open source hardware & software system
Subject area	• Engineering and materials science • Educational tools and open source alternatives to existing infrastructure • Additive manufacturing
Hardware type	• Mechanical engineering and materials science • Other [Hybrid manufacturing]
Closest commercial analog	No commercial analog is available.
Open source license	CC BY 4.0
Cost of hardware	∼275€ (225€ + 50€ waterjet cut parts aproximated price)
Source file repository	https://dx.doi.org/10.17632/jvmd5p3rtx.2

## 1 Hardware in context

For several decades, the impact of additive manufacturing has been increasingly felt across various industrial sectors. The constant advancements and the multitude of fields in which it has the potential to revolutionize the way we work, design, and produce, strengthen our belief that it could be at the forefront of a new industrial revolution.

Among the various existing additive manufacturing technologies, there is one that particularly appeals to small and medium-sized companies, as well as individuals: the Fused Deposition Modeling (FFF) process. Its accessibility with a low initial investment (in the range of a few hundred euros [1]) as well as its easy implementation make it the first choice for entities with limited resources. However, FFF has some drawbacks: the use of polymers with relatively limited performance, as well as lesser surface finishes and dimensional accuracy. An approach to address these inherent problems of additive manufacturing by material deposition would be to combine it with a subtractive process such as CNC machining. FFF additive manufacturing allows for the creation of complex geometries but is limited in terms of accuracy and materials. Conversely, machining guarantees higher levels of accuracy on very high-performance materials but requires a high level of expertise in return. The combination of additive and subtractive manufacturing could help offset the limitations of each process by achieving a certain balance.

The first experiments in hybridizing additive and subtractive technologies began in the mid-1990s [2] and mainly focus on a DED-CNC combination [3], namely, high added-value technologies for high-performance materials [4]. Regarding FFF/CNC hybridization, some manufacturers have recently successfully brought to market specially dedicated machines.^1^ In research area, the combination of those technologies has been widely practiced [5] and the process is usually carried out using an existing platform (CNC) by mounting an extruder in place of a cutting tool [6], [7], [8], [9], [10]. However, papers can be found where multi-axis hybrid process is carried out using robots [11], [12], [13], specific hybrid machines designs are proposed and made available [8], [14] or where a 3D printer is directly used for hybrid purposes [15]. Some studies specifically dedicated to this discipline are starting to be published and address the various challenges raised by this new process: the integration of the fused layer modeling process into a CNC architecture designated for subtractive manufacturing [6], methods to remedy the lack of inter-layer adhesion after machining phases [16], the benefits in terms of productivity and the savings on raw materials [9], the correlation between material deposition strategy and the machinability of the green part in 3D MIM-like printing [9]. The emergence of new machines capable of implementing various processes is starting to appear, but public adoption is slow. This can be attributed to the need to address several technical challenges [4], due to high investment costs and potential industrial patents slowing down developments. In light of this, the distribution of free open-source hardware (FOSH) has proven to be particularly promising for the development of customized scientific tools [17] and the present work is directed in this regard. The authors' motivation is to help democratize access to advanced manufacturing tools in a field where cost and technical complexity can be significant barriers. Indeed, the various technical solutions listed in the literature can have the disadvantage of imposing relatively heavy economic or technical constraints. Mounting a simple machining spindle on an easily accessible FFF printer, combined with a suitable hybridization method, could pave the way for wider adoption of the hybrid process by the general public and research area.This paper presents an Open Source & low-cost solution for FFF/CNC hybridization accessible to the widest audience, especially to entities with relatively modest means. The equipment proposed here is based on the work of the British company E3D published on their blog^2^ at the end of 2020. The E3D team presents a complete set with a “DIY” spindle and a post-processor for machining with AutoDesk Fusion 360. The work presented in this article improves upon the spindle and offers two entirely Open Source software alternatives. The first is hybridization via the separate use of a CAM software (for users proficient in machining) with the development of a post-processor in the FreeCad software. The second alternative is the automatic machining of the part via “SuperSlicer”. This latter option allows any user to perform the machining of parts without prior knowledge in this discipline. We will see that depending on the chosen solution, the manufacturing process will differ: using CAM allows for “sequential” hybridization, while using SuperSlicer will enable “layer by layer” hybridization. The work presented also includes a stabilization method for the spindle (specific to E3D's “ToolChanger” system) as well as a characterization of the surface roughnesses obtained through each process: regular FFF printing, layer by layer hybrid, or sequential hybrid.

We begin by detailing the design of the machining spindle and the various stages involved in its assembly and construction. The various software solutions for implementing the hybrid process are then briefly presented. Finally, the results of experiments and tests carried out on the spindle to characterize its performance with two different hybrid solutions are presented.

## 2 Hardware description

**Fig. 1:**
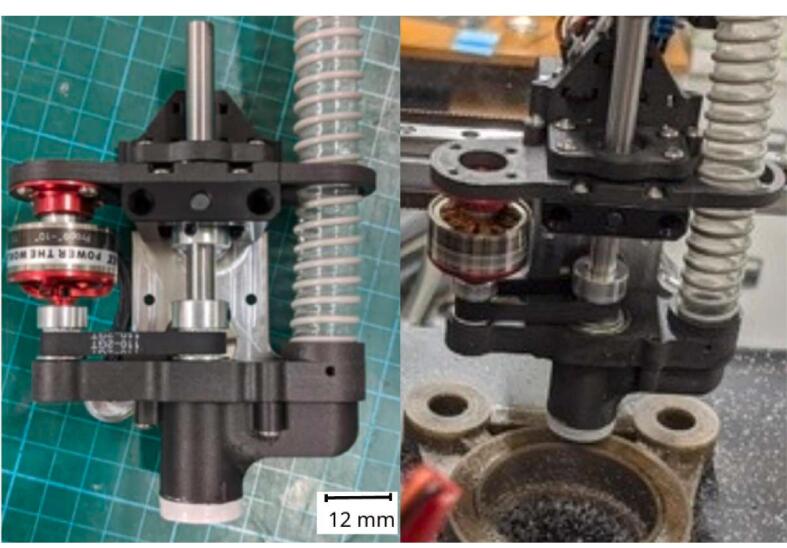
ASMBL substractive tool, Greg_The_Maker, Thingiverse thing: 4,206,827.

The spindle made available^3^ by E3D (Fig. 1) features a design intended to be easily producible, primarily targeting the general public (“Makers”). The system mounts on their multifunctional machine “ToolChanger” and consists of a brush less motor with its ESC (Electronic Speed Controller), a chuck, as well as a set of pulleys and a belt. The remaining parts can be printed with any additive manufacturing machine.

The concept is highly intriguing; however, some deficiencies become apparent when it comes to intensive use of the equipment. The authors of the text revisited the design for two main reasons:

- Some choices made in the original design compromise the system's rigidity, and therefore its ability to machine parts accurately.
- The spindle should be made with durable materials that can withstand the stresses imposed by subtractive phases. The system designed in the context of this article is intended to be manufactured from an aluminum alloy using stacked cut plates.

**Fig. 2:**
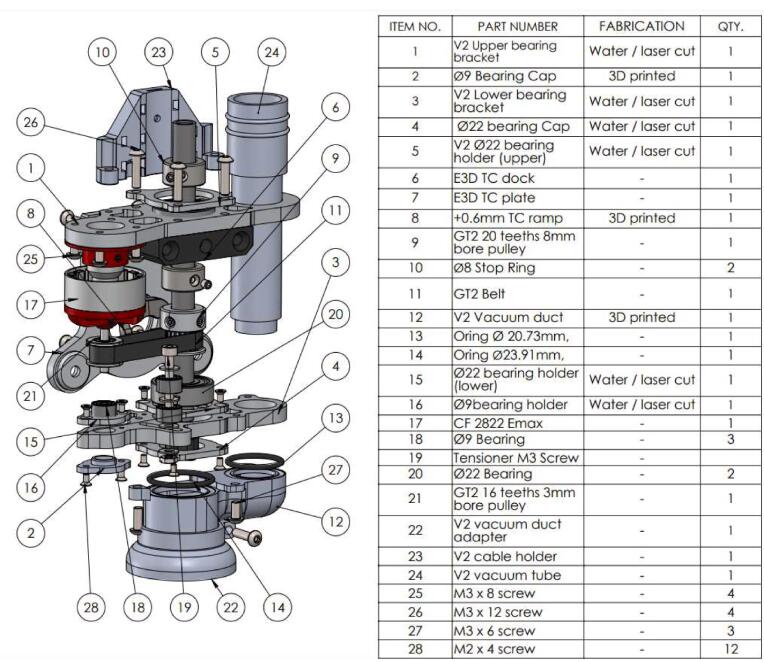
Exploded view of the system (belt tensioner version).

The optimization that was carried out starts with a general redesign to stiffen the assembly, and the new design is oriented to allow for the parts to be cut (waterjet/laser) from an aluminum alloy (Fig. 2). The machining spindle remains feasible at a very low cost and requires no CNC operations. Additional modifications are made to the system, including the improvement of chip extraction, the addition of a belt tensioner (optional), and a clamping system for the spindle.

### 2.1 Modifications to the original design

**Fig. 3:**
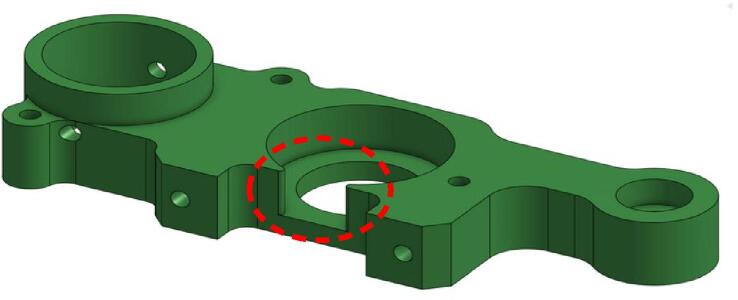
Opening of the bearing housing on the “Lower bearing bracket” part.

The first modification concerns the lower part of the spindle, at the level of the Lower Bearing Bracket piece. The original part has a significant opening at the bearing housing, as indicated in Fig. 3. This choice may have been made for simplicity, to avoid offsetting the spindle's axis so that it matches the axis of the machine's other printing systems. The integration of the other parts and the spindle itself is also made easier. However, it's at this location that the part, and the system as a whole, exhibit their fragility and lack of rigidity, which is undesirable for a machining spindle.

**Fig. 4:**
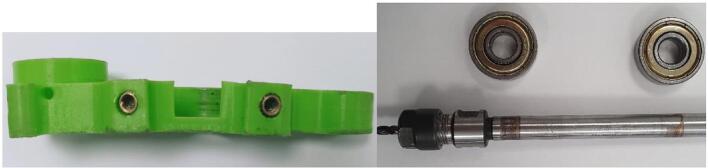
Deformation on the original lower bearing bracket.

The photos in Fig. 4 were taken after dismantling the spindle following 2 h of use. We observe that the Lower bearing bracket piece has deformed, which resulted in the release of the bearing and a misalignment of the rotation axes of the motor and the spindle.

The Lower Bracket Bearing and Upper Bracket Bearing parts have been modified by moving the bearing positions and incorporating recesses to limit the weight of the whole. The design proposed here is not intended to be 3D printed; implementing functional dimensioning with mechanical engineering standards is relevant. The clamping force applied to the bearings is relatively low. Their assembly can be done manually using a mallet or a vice, in line with an implementation with simple means accessible to all. The tolerance classes from the ISO system [18] chosen for the two bearings are a 22 H8 diameter for the spindle axis bearing and a 9 H9 diameter for the motor bearing. This corresponds to **⌀** 22 mm (+0.033/−0) and **⌀** 9 mm (+0.036/−0) respectively. Therefore, a machine reamer mounted on a drill press with precise assembly can meet these specifications.

**Fig. 5:**
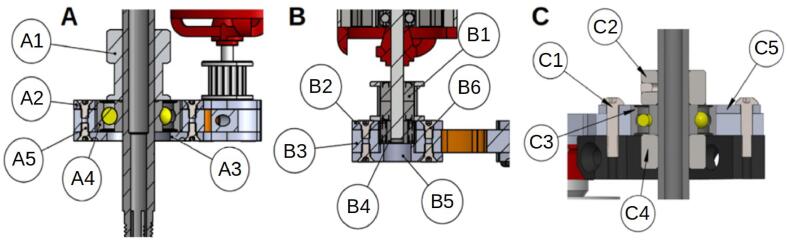
Cross-sectional views of the bearing housings: lower bearing bracket (A, B) and upper bearing bracket (C).

The design by E3D has the advantage that the bearings are held in translation by the frame and a pulley. The modification proposed here is achieved through stacking plates. Holding parts as well as retaining rings are placed to keep the bearings in their housings (Fig. 5). The parts are made by cutting. The precision isn't sufficient, and the bearing bores are defined on the drawing with an excess of 0.5 mm in radius to be adjusted with a reamer mounted on a drill press.

On the Fig. 5. A we can see that the main bearing (A4) of the lower bearing bracket is held in its housing by the 20 T pulley (A1) and a cover plate (A3). A holding plate (A2) is placed on the upper side, it helps to increase the bearing surface held in the housing. The entire assembly is held by short M2 screws (A5). Fig. 5B, the same principle is applied at the level of the motor shaft bearing (B4). The latter is held by a holding piece (B2), and a 3D printed cover plate (B5), the translation is blocked by the pulley (B1). The system is mounted via M2 screws (B6) on the lower bracket bearing (B3). Fig. 5C shows how on the upper bracket bearing, the bearing (C3) is held in place by two stop rings (C2, C4). A holding plate (C5) is mounted on the assembly with four M3 screws (C1).

### 2.2 Modification of the chip collecting system and implementation of belt tensioner

**Fig. 6:**
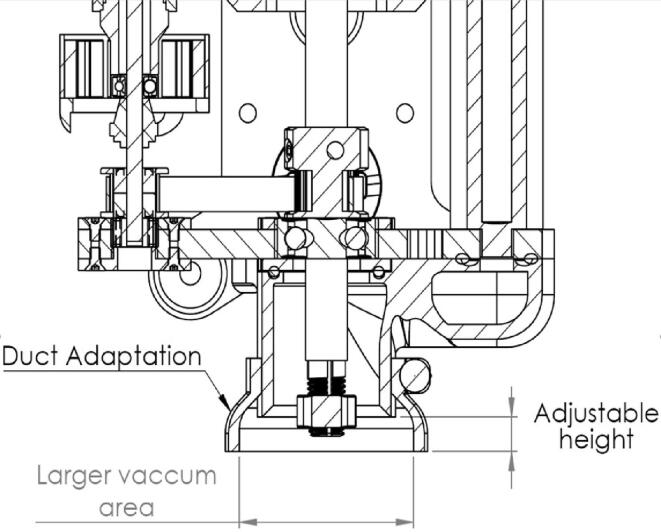
Chip collection system.

Machining as practiced here does not necessarily require lubrication. However, the workspace, the slides, and other mechanical elements are sensitive to the pollution generated by subtractive phases. A suction system is present in the original design, but its efficiency is limited when the cutting tool exceeds a certain length. The suction area is too far from the machining area. To remedy this problem, an additional part is designed, “Duct adaptation” (Fig. 6). This part fulfills two basic functions: adjust the height to bring the suction closer to the cutting area and create a diverter to increase the surface on which the suction takes place. To seal the system, grooves and O-rings are placed.

**Fig. 7:**
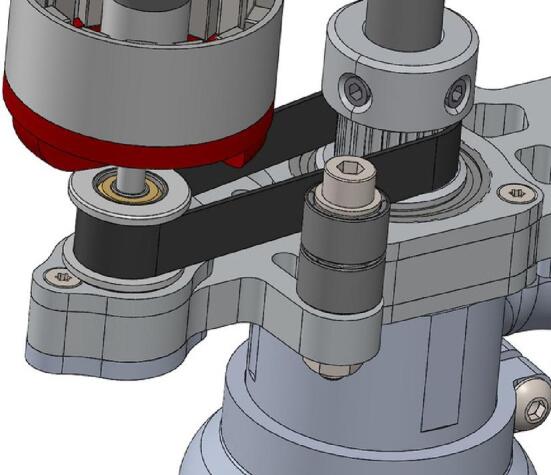
Lower Bracket bearing with belt tensioner.

The spindle is powered by a brushless motor typically used for drones. This type of motor offers relatively high rotation speeds (up to 20,000 RPM) for a low weight and small size. This makes it an interesting option for machining parts printed with soft materials like PLA or ABS, which are commonly used in FFF 3D printing. The maximum speed of the spindle, ±10,000 rpm, might be insufficient for small diameter mills. If we take the example of a mill with a diameter D of 1.5 mm in HSS (High-Speed Steel) and a maximum rotational speed N captured at 10,800 rpm to machine a carbon fiber-loaded polymer of type ABS or PETG, a cutting speed Vc = N × π × D/1000 of 50.8 m/min would be achieved. We are far from reaching the cutting speeds (90 -300 m/min) for machining polymers [19], [20], [21]. A version of the “Lower bearing bracket” piece with a belt tensioner system was also designed in case the user wishes to change the reduction ratio by modifying the diameter of the pulleys (Fig. 7). Thus, if rotating components were to be changed, the belt tension would be adjustable. The tensioner, made of an assembly of 2 bearings tightened on the inner rings, slides in a groove to adjust the belt tension. A test was conducted by changing the pulleys (36/18 teeth instead of 20/16 teeth) to increase the spindle rotation speed and thus obtain a reduction ratio of 2 (compared to 1.25 for the original spindle) allowing the system to reach speeds around 26,000 RPM.

### 2.3 Limitations of waterjet cut plates use

**Fig. 8:**
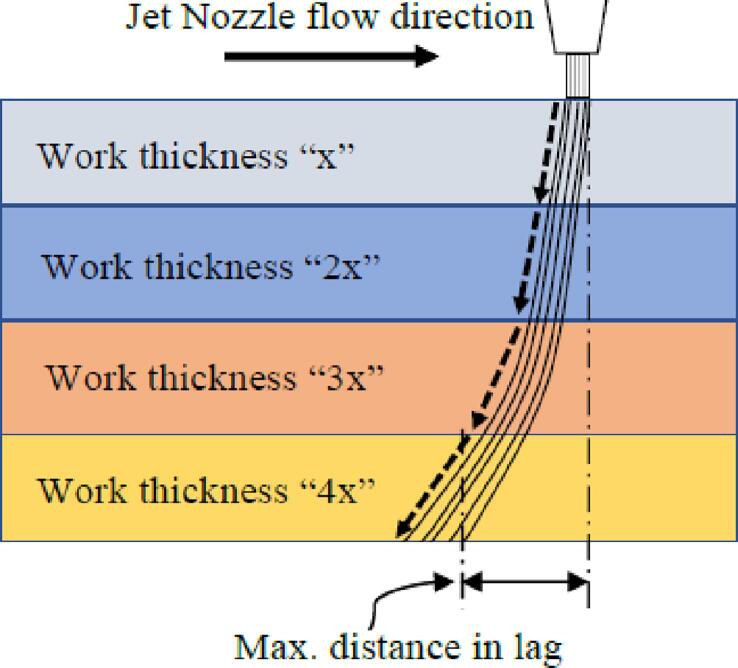
Deviation in jet cutting intensity.

**Fig. 9:**
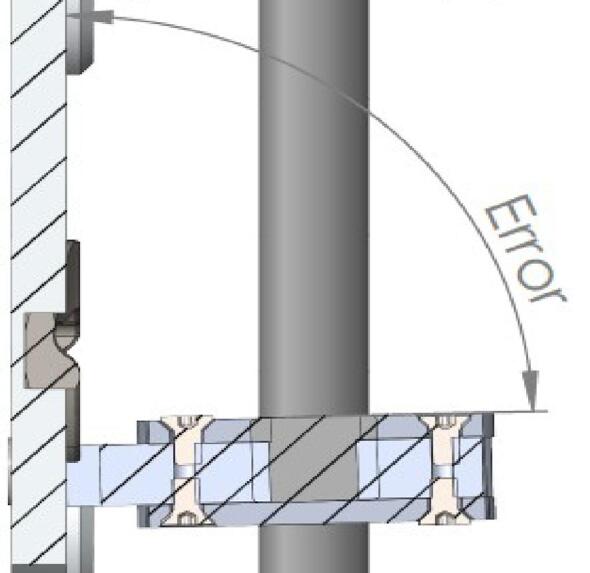
Alignment defect on the spindle.

Apart from the impact of the operator's ability to correctly carry out the bores and various drillings, the precision of the cut parts can be problematic when it comes to functional surfaces. These often have a draft (Fig. 8) or a poor surface finish [22] which can lead to an alignment defect of the spindle through the Lower bracket bearing. (Fig. 9). To remedy this defect, a surfacing pass on the “left” faces may prove to be necessary.

### 2.4 Stabilization of the tool changing system

**Fig. 10:**
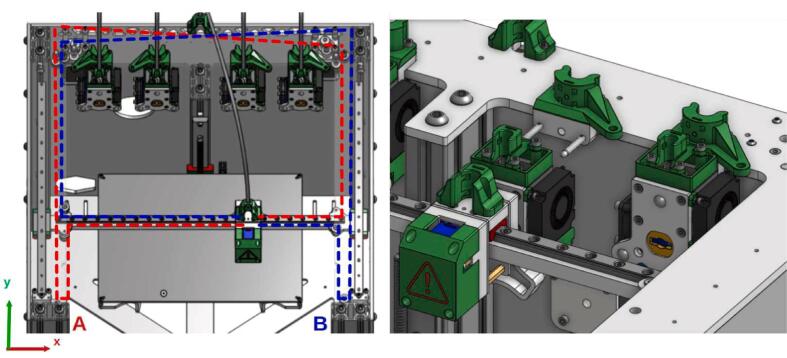
Tool changer Core XY motion system.

The tool holder system of the ToolChanger has also been modified. The original system is detailed in this section to understand this modification. The machine is equipped with a Core XY where two belts (A and B, Fig. 10) and two synchronized motors allow the movement of the tool carrier on the horizontal plane. The tools are “docked” on the chassis. During loading or unloading, the carrier moves and positions itself in front of the desired tool.

**Fig. 11:**
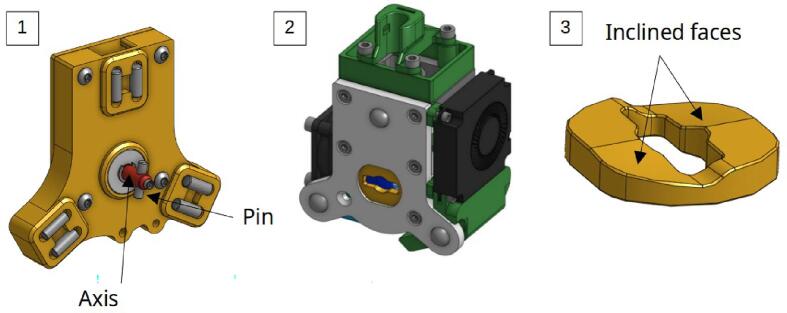
Tool carrier (1), tooling system (2), grooved ramp plate 3.

The entire system is illustrated Fig. 11. The tool/carrier connection is made using a Maxwell kinematic coupling [23]: The tool plate (2) is placed against the carrier (1), the elimination of the 6 degrees of freedom ensures positioning. When the carrier (1) is engaged at the tool's position, the pinned axis of the carrier passes through the opening of the grooved plate (3) and makes a 90° rotation: the pin slides on the inclined walls of the grooved plate, causing with it a translation of the axis, and the whole is held in place thanks to the force of a compression spring placed at the stop of the pinned axis.

**Fig. 12:**
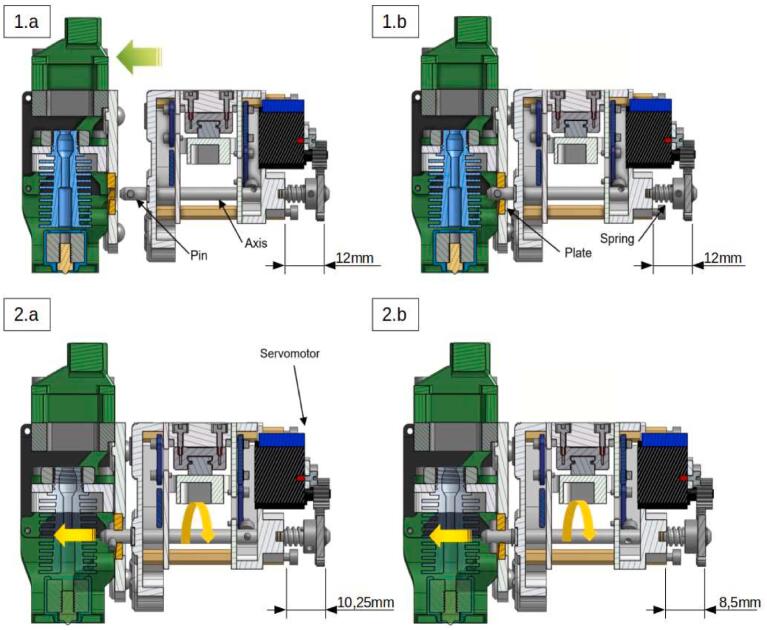
Tool changing process step by step.

The tool change process is schematically represented in Fig. 12:

- 1.a: Approach phase of the carrier.
- 1.b: Engagement. At this stage, the carrier system is in its “open” position: the pinned axis has an angle of 0° and it does not translate.
- 2.a: Start of locking. The axis begins a rotation to clamp the system, the pin at its end slides on the walls of the grooved plate ramp and the axis translates in its bore.
- 2.b: Locking. The axis has made a 90° rotation and has moved by 3.5 mm. The spring is compressed and holds the assembly; the carrier and the tool can be withdrawn.

**Fig. 13:**
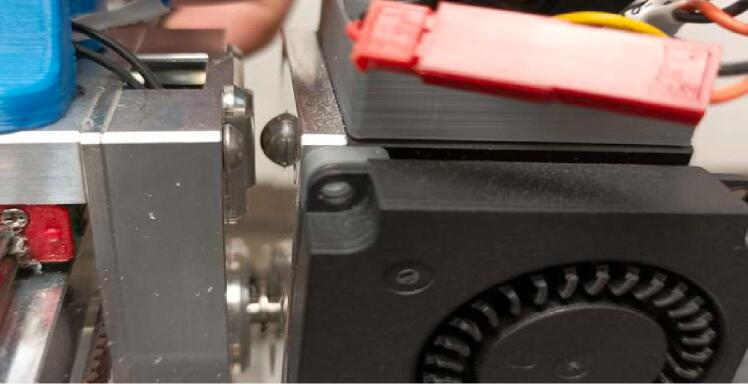
Detachment of the tool system.

Although the E3D system is perfectly suited for 3D printing, it is not compatible with machining and the cutting forces inherent to it. The clamping provided by the pressure imposed by the compressed spring does not allow the spindle to be held during subtractive phases. A simple thumb pressure on the system is enough to move the axis in translation and separate the carrier from the tool system as shown in Fig. 13.

**Fig. 14:**
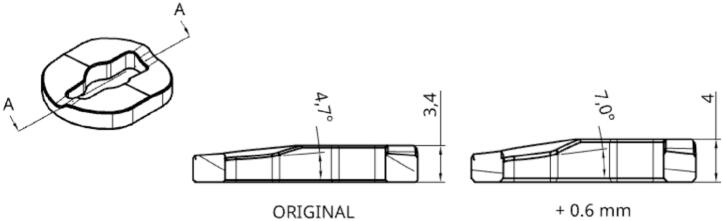
Modified grooved plate ramp.

**Fig. 15:**
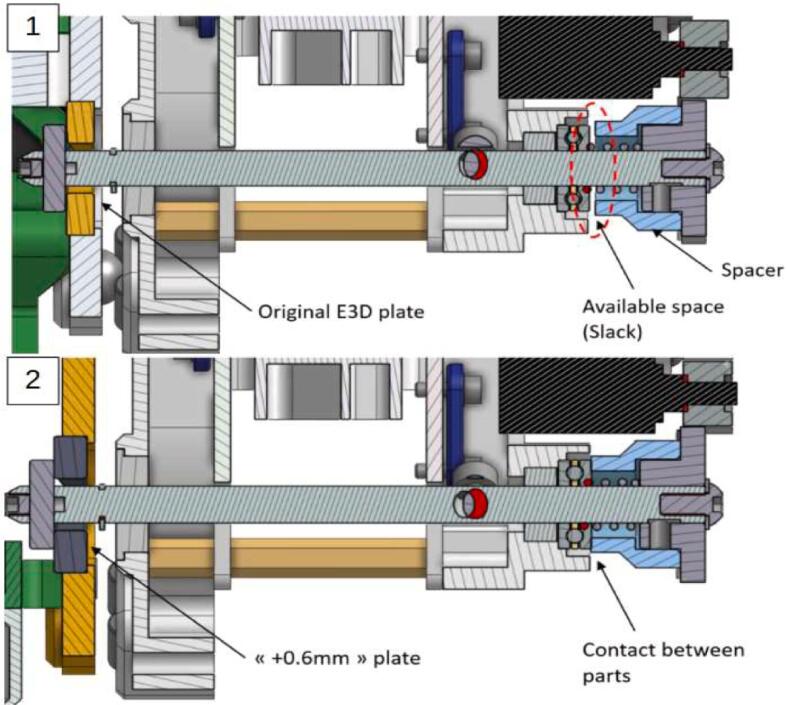
Locking with and without the new grooved plate ramp.

To clamp the spindle and prevent any vibration during machining while leaving the printing tools intact, a thicker grooved plate ramp (Fig. 14) is designed and placed on the machining systems to increase the translation of the axis only for these tools. A spacer is then placed on the carrier's axis between the pinion and the ball stop facing it. With an adjusted length, this spacer allows the assembly to be clamped by preventing any movement of the axis once it completes its translation. Fig. 15 illustrates the situation of the system when locking a tool with a standard grooved plate ramp (original assembly) once the spacer is mounted on the carrier. When loading the machining spindle (equiped with a modified grooved plate ramp), the carrier's axis performs an additional 0.6 mm translation and the spacer comes into contact with the ball stop (2). The system is clamped, no translation of the lock axis and therefore of the tool is possible. A clearance of 0.6 mm between the ball stop and the spacer will be available for all other tool system (1).

### 2.5 Software for hybrid fabrication

At present, there are few software initiatives aimed at correlating FFF and CNC fabrications in an automated way to give rise to a native hybrid process. This is partly because the development of computer tools is linked to the emergence of dedicated equipment and vice versa. Even if E3D offers a hybrid solution with Fusion 360 software, it is not Open Source and requires machining knowledge. We propose two solutions based on free software to implement two different hybridization methods:

− Sequential hybridization (FreeCad): machining the entirety or part of a piece in multiple subtractive sequences, depending on the pass depth allowed by the tool or geometry. Machining phases occur throughout the printing process. This method requires machining skills. Currently, the only softwares allowing this type of process are NX Siemens and Fusion 360 because they integrate virtual workspaces for machining and FFF 3D printing. The authors provide a post-processor to carry out the programming of the subtractive phases on the Open Source software FreeCad. However, the method is not automated, and the codes corresponding to machining passes must be manually integrated into the Gcode. Also, machining and printing are not programmed in the same environement.
− Automatic layer-by-layer hybridization: automated machining carried out at each deposited layer. Currently, the only solution to implement this hybrid process is the slicer “SuperSlicer” which has integrated this functionality. This method currently only allows contouring operations, unlike sequential hybridization, but it is a native hybrid that requires no machining skills. It is therefore more accessible.

**Fig. 16:**
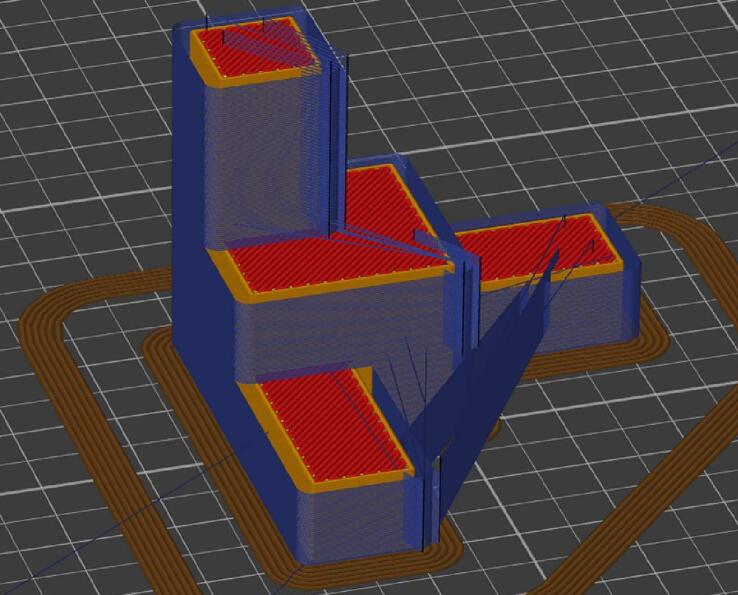
“Layer by layer” hybrid with SuperSlicer: machining paths in blue.

**Fig. 17:**
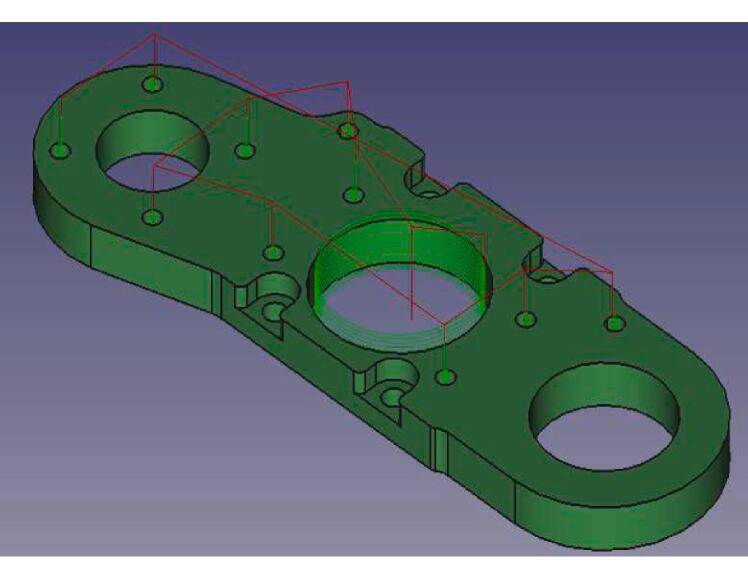
Sequential hybrid of functional zones.

The implementation of layer-by-layer hybridization (Fig. 16) and sequential hybridization (Fig. 17) are detailed in the “Operation instructions” section.

## 3 Design files summary

All CAD files, summarized in the table below, can be downloaded from the Mendeley repository (https://dx.doi.org/10.17632/jvmd5p3rtx.2). A general assembly file as well as each individual part are available in STEP format. CAD files for standard components are not included except for the ER extension shaft, pulleys, and stop rings.

**Table:** 

Design file name	File type	Manufacturing	Open source license	Location of the file
Assembled spindle	CAD	−	CC BY 4.0	Mendeley repository
V2 Upper bearing bracket	CAD	Laser/Waterjet cutting	CC BY 4.0	Mendeley repository
Ø9 Bearing cap	CAD	3D printing	CC BY 4.0	Mendeley repository
V2 Lower bearing bracket	CAD	Laser/Waterjet cutting	CC BY 4.0	Mendeley repository
Ø 22 Bearing cap	CAD	3D printing	CC BY 4.0	Mendeley repository
Ø 22 Bearing holder (upper)	CAD	Laser/Waterjet cutting	CC BY 4.0	Mendeley repository
E3D TC dock	CAD	−	−	Mendeley repository
E3D TC plate	CAD	−	−	Mendeley repository
+0.6 mm TC ramp	CAD	3D printing (Resin)	CC BY 4.0	Mendeley repository
GT2 20 teeths 8 mm bore pulley	CAD		CC BY 4.0	Mendeley repository
Ø 8 Stop ring	CAD		CC BY 4.0	Mendeley repository
V2 vacuum duct	CAD	3D printing	CC BY 4.0	Mendeley repository
Ø 22 Bearing holder (lower)	CAD	Laser/Waterjet cutting	CC BY 4.0	Mendeley repository
Ø 9 Bearing holder	CAD	Laser/Waterjet cutting	CC BY 4.0	Mendeley repository
GT2 16 teeths 3 mm bore pulley	CAD	−	−	Mendeley repository
Vacuum duct adapter	CAD	3D printing	CC BY 4.0	Mendeley repository
V2 Cable holder	CAD	3D printing	CC BY 4.0	Mendeley repository
V2 Vacuum tube	CAD	3D printing	CC BY 4.0	Mendeley repository
V2 Lower bearing brackets- Drilling jig	CAD	3D printing	CC BY 4.0	Mendeley repository
V2 Upper bearing bracket- Drilling jig	CAD	3D printing	CC BY 4.0	Mendeley repository
Drilling jigs (others)	CAD	3D printing	CC BY 4.0	Mendeley repository
+0,6 mm ramp matrix	CAD	3D printing	CC BY 4.0	Mendeley repository

## 4 Bill of materials summary

Standard components can be purchased through the links provided in the table below. The links provided are valid as of the publication date of this article.

**Table:** 

Designator	Component	Qty	Cost	Total cost	Source of materials
−	Blank Tool Plate & Dock Kit	1	69,63€	69,63€	https://e3d-online.com/products/blank-tool-plate-dock-kit
GT2 20 teeth 8 mm bore pulley	GT2 20 teeth 8 mm bore pulley	1	2,18€	2,18€	https://www.ebay.co.uk/sch/i.html?_from=R40&_trksid=m570.l1313&_nkw=20T+GT2+Pulley+8mm+Bore&_sacat=0&ff3=4&pub=5575249786&toolid=10001&campid=5337999268&customid=&mkevt=1&mkcid=1&mkrid=710-53481-19255-0&ufes_redirect=true
Ø 8 Stop ring	Ø 8 Stop ring (kit)	1	−	12€	https://www.amazon.de/-/en/Mengger-Position-Regulator-Positioner-Woodworking/dp/B07ZHBP8HX/ref=sr_1_7?crid=2YIQGHB8OMBNZ&keywords=dia.+8+stop+ring&qid=1682794962&s=diy&sprefix=dia.+8+stop+ring%2Cdiy%2C73&sr=1-7
GT2 Belt	GT2 55 teeths (110 mm) closed belt	1	2,70€	2,70€	https://www.ebay.co.uk/sch/i.html?_from=R40&_trksid=m570.l1313&_nkw=55T+%28110mm%29+Closed+Loop+GT2+Belt&_sacat=0&ff3=4&pub=5575249786&toolid=10001&campid=5337999268&customid=&mkevt=1&mkcid=1&mkrid=710-53481-19255-0&ufes_redirect=true
Ø 20.73O-ring	Ø 20.73O-ring (kit)	1	6,66€	6,66€	https://www.123roulement.com/joints-OR-17.17X1.78-AU94
Ø 23.91O-ring	Ø 23.91O-ring (kit)	1	7,33€	7,33€	https://fr.rs-online.com/web/p/joints-et-joints-toriques/1964742
CF 2822 Emax	CF 2822 Emax	1	20€	20€	https://www.ebay.co.uk/sch/i.html?_from=R40&_sacat=0&ff3=4&pub=5575249786&toolid=10001&campid=5337999268&customid=&mkcid=1&mkrid=710-53481-19255-0&ufes_redirect=true&_nkw=1200kv+cf+2822+emax+brushless+motor&rt=nc&LH_PrefLoc=3
Ø 9 Bearing	MR93ZZ bearing	1	4€	4€	https://www.ebay.co.uk/sch/i.html?_from=R40&_trksid=m570.l1313&_nkw=MR93ZZ+Bearing&_sacat=0&ff3=4&pub=5575249786&toolid=10001&campid=5337999268&customid=&mkevt=1&mkcid=1&mkrid=710-53481-19255-0&ufes_redirect=true
Ø 22 Bearing	608ZZ bearing	2	2€	4€	https://www.ebay.co.uk/sch/i.html?_from=R40&_trksid=p2380057.m570.l1313.TR11.TRC1.A0.H0.X608zz+bearing.TRS0&_nkw=608zz+bearing&_sacat=0&ff3=4&pub=5575249786&toolid=10001&campid=5337999268&customid=&mkevt=1&mkcid=1&mkrid=710-53481-19255-0&ufes_redirect=true
−	M2 screws (kit)	1	18€	18€	https://www.amazon.fr/HVDHYY-Cylindrique-Dassortiment-Rangementpour-Professionnels/dp/B0BKGSNPDP/ref=sr_1_20?__mk_fr_FR=%C3%85M%C3%85%C5%BD%C3%95%C3%91&crid=IXHDBFSDRKB&keywords=M2%2Bscrews&qid=1682796696&s=hi&sprefix=m2%2Bscrews%2Cdiy%2C74&sr=1-20&th=1
−	CPAP pipe	1	33,90€	33,90€	https://www.intushealthcare.com/product/4ft-lightweight-travel-tubing/
−	Emax motor ESC	1	13,5€	13,5€	https://www.ebay.co.uk/sch/i.html?_from=R40&_trksid=m570.l1313&_nkw=MAX+12a+SimonK+Brushless+ESC&_sacat=0&ff3=4&pub=5575249786&toolid=10001&campid=5337999268&customid=&mkevt=1&mkcid=1&mkrid=710-53481-19255-0&ufes_redirect=true
GT2 16 teeths 3 mm bore pulley	GT2 16 teeths 3 mm bore pulley	1	3€	3€	https://www.ebay.co.uk/sch/i.html?_from=R40&_trksid=m570.l1313&_nkw=16T+GT2+Pulley+3mm+Bore&_sacat=0&ff3=4&pub=5575249786&toolid=10001&campid=5337999268&customid=&mkevt=1&mkcid=1&mkrid=710-53481-19255-0&ufes_redirect=true
Collet chuck	C8-ER8 100L Collet Chuck	1	13,5€	13,5€	https://www.ebay.co.uk/sch/i.html?_from=R40&_trksid=m570.l1313&_nkw=C8-ER8+100L+Collet+Chuck&_sacat=0&ff3=4&pub=5575249786&toolid=10001&campid=5337999268&customid=&mkevt=1&mkcid=1&mkrid=710-53481-19255-0&ufes_redirect=true

## 5 Build instructions

### 5.1 Realization of the parts

**Fig. 18:**
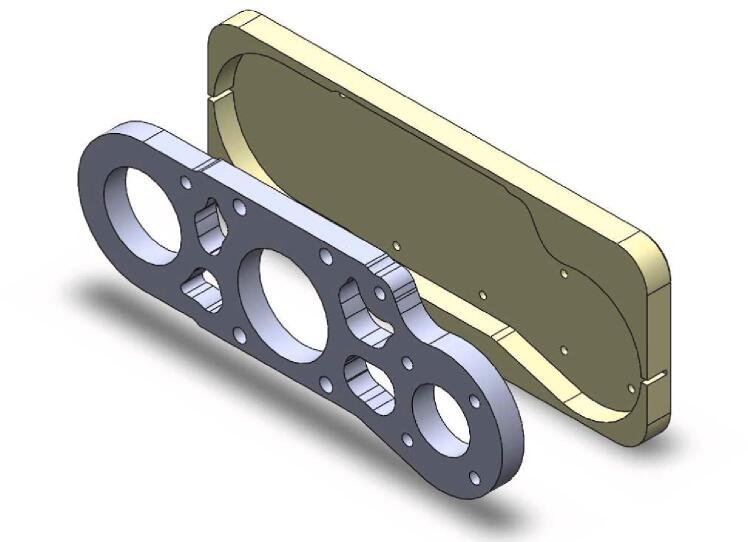
Drilling jig for the Upper bearing bracket.

The drillings and threadings for screws cannot be made using cutting; hence, a series of positioning jigs has been designed. They assist the operator in marking the position of each drilling (Fig. 18).

All machining operations are described below. Some specific tools/machines are required:

− Drills Ø 2.5 mm, Ø 1.6 mm & Ø 3.5 mm.
− Taps M3x0.5 & M2x0.4.
− Reamers Ø 22 H7 & 9H7.
− Small diameter 45° chamfering tool.
− Drill press with Morse taper head.

**Fig. 19:**
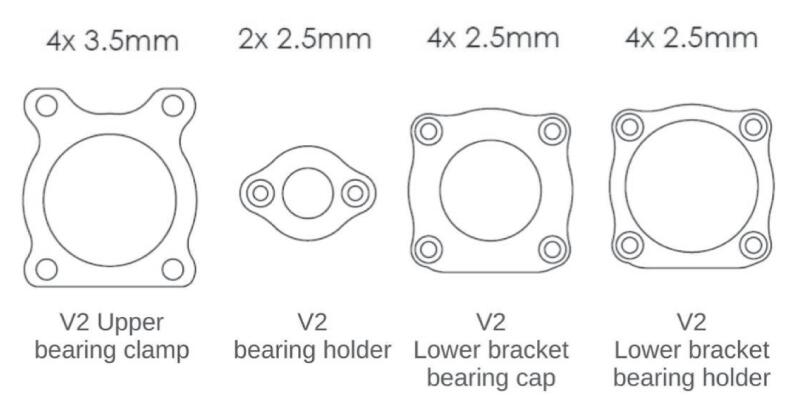
Auxiliary parts.

After deburring, the parts shown in Fig. 19 are placed in their jigs, center-pushed, and drilled. The 45° chamfers are used to countersink the screw heads.

**Fig. 20:**
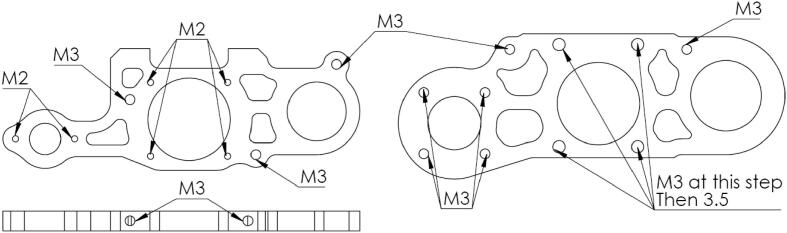
Drilling of the bearing brackets.
− Assemble the “Ø 22 Bearing holder” parts (upper) onto the Upper bearing bracket with M3 screws, and then assemble the “ Ø 22 Bearing holder” (lower) and “ Ø 9 Bearing holder” onto the lower bearing bracket with M2 screws.
− Mount the assembly in the vise with the bearing holder parts facing downward (Fig. 21): the reamer should center on the faces of the Upper and Lower bearing brackets.

− Perform the drillings and threadings on the Upper and Lower bearing brackets as shown in Fig. 20. Note that 4 of the drillings in the Upper bearing bracket should be drilled to M3 at this stage. Perform the lateral M3 drillings on the Lower bearing bracket (these drillings define the alignment of the spindle axis, special attention should be given to this operation).

**Fig. 21:**
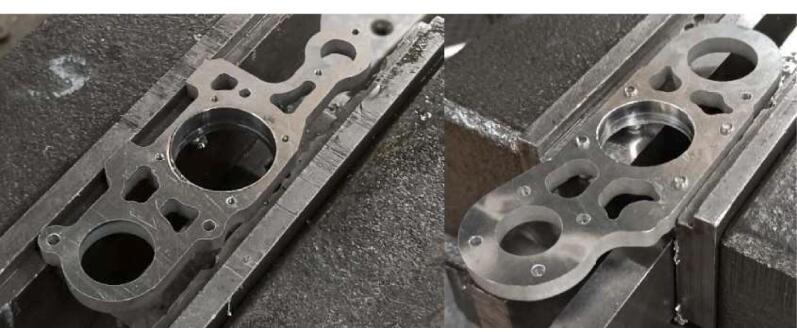
Reaming of the bearing brackets.
− After centering the assembly, perform the Ø 22 bores. Repeat the operation for Ø 9. Once machining is complete, drill Ø 3.5 mm holes in place of the four temporary M3 holes previously indicated on the Upper Bearing bracket.

### 5.2 Assembly

**Fig. 22:**
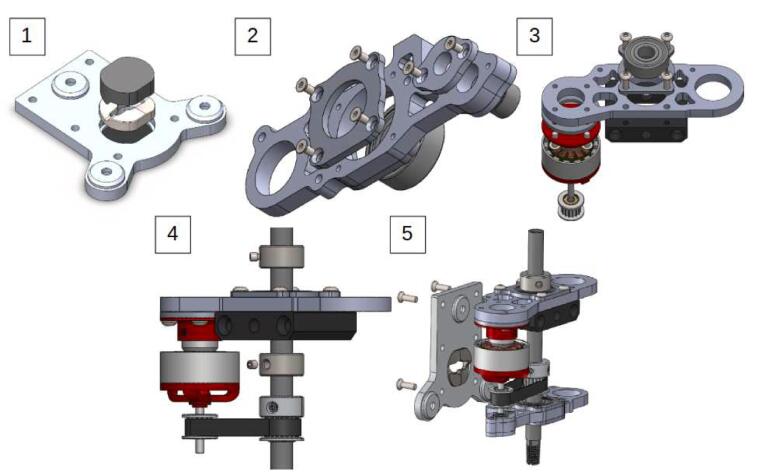
Assembly of the spindle (no tensioner version).

The assembly of the spindle is illustrated in Fig. 22. The assembly of the suction system is relatively simple and is not detailed in the diagrams. The necessary tools are: Allen key set/ Cyanoacrylate glue/ Workshop vice.

− Remove the original grooved plate ramp from the TC plate. The plates are glued with cyanoacrylate glue, and the use of acetone may be necessary. Mount the + 0.6 mm grooved plate ramp using the matrix tool.
− Assemble the Ø 9 Bearing cap and Ø 22 Bearing holder (lower) with the lower bearing bracket using M2 screws. Mount the Ø 22 bearing.
− Repeat the previous step with the upper part of the spindle. Assemble the motor and the 16-tooth pulley and mount the assembly on the upper bearing bracket. It may be necessary to disassemble the motor for attachment with the 4 M3 screws.
− Mount the 20-tooth pulley and the Ø 8 stop rings on the collet chuck. Place the belt. The Ø 8 stop rings should lock the collet chuck in translation and are placed on either side of the upper Ø 22 bearing.
− Slide the lower assembly into the spindle axis. Mount the TC Plate on the assembly with the original M3 screws. Adjust the height of the pulleys and Ø 8 stop rings, and check the tightness. For the version with a belt tensioner, place the two Ø 9 bearings on top of each other with a washer (x3 M3 washers) between the inner rings of the bearings and the bearing surfaces as previously shown in Fig. 7.

## 6 Operation instructions

### 6.1 Subtractive phases programming

FreeCad software includes computer-aided manufacturing (CAM) functionality for machining.^4^ Thanks to the post-processor developed by the authors (available on the Mendeley repository), subtractive phases can be programmed and executed with the Tool Changer at the end of printing or by manually including them in the Gcode at the desired height. To perform machining, two conditions must be met besides having CNC programming knowledge: correctly positioning the piece in the workspace (similar to the slicer) and using a standard 3D file (not an STL).

**Fig. 23:**
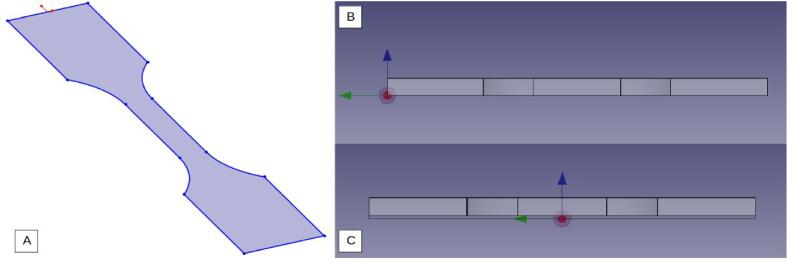
CAD of the tensile test specimen (A), positioning without adjustments (B), corrected positioning (C).

**Fig. 24:**
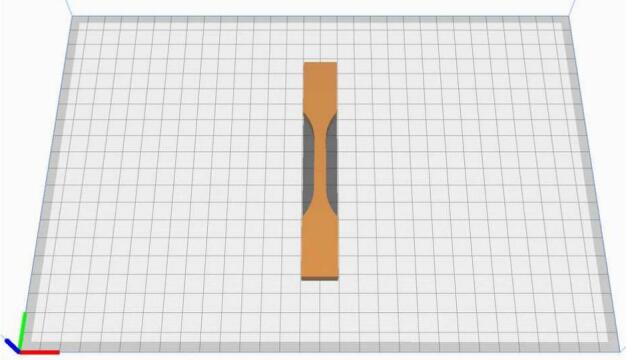
Automatic positioning in Cura slicer.

In conventional computer-aided design and manufacturing (CAM) software, a user-chosen program origin point is used to define the position of the workpiece in space. By default, FreeCad will place the program origin at the workpiece origin (the origin of the drawing during the CAD phase), while slicers (such as Cura used in their work) will position the workpiece relative to the center of its convex hull in the middle of the build plate. Therefore, it is necessary to ensure that the positioning of both programs corresponds, and if necessary, modify the program origin in FreeCad. This issue is illustrated Fig. 23. On a tensile test specimen whose workpiece origin point from CAD (A) is located on the upper part, FreeCad will place the program origin point for machining (B) there. An action is needed to align the positioning of Cura, Fig. 24 and FreeCad. In this example, we move the program origin to the center of the workpiece, as shown in Fig. 23(C), and raise the workpiece by the thickness of the raft if it is configured in Cura.

To enable RepRap to understand the FreeCad code, a Grbl root post-processor was designed, and the following actions were taken:

1. Modification of the “Preamble”: activation of Mesh Compensation.
2. Modification of the “Postamble”: unloading the tool at the end of the operation.
3. Activation of tool change at the beginning of the operation.
4. Deactivation of commands dedicated to the spindle.
5. Automatic offset of the program origin point (X −150, Y −100) to match the Tool changer's machine origin point (bottom left, Fig. 24).

In the context of automated “layer-by-layer” hybridization, the SuperSlicer software, which has integrated the “Milling post process” functionality, will allow contouring operations to be performed based on the path taken by the nozzle. Once the size of the milling cutter is specified, the software provides the option to adjust the feed rate as well as the amount of additional material to be printed and milled.

### 6.2 Modification of the tool CNC loading macros

**Fig. 25:**
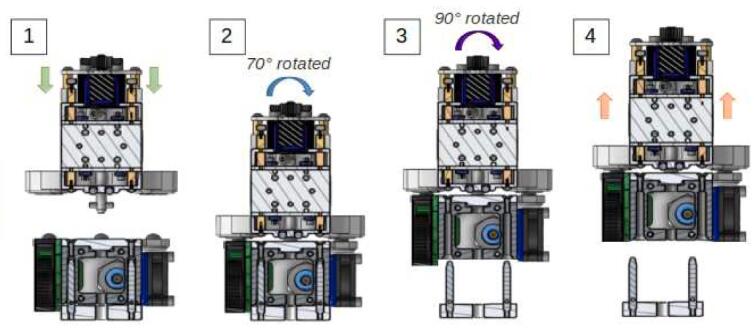
CNC tool loaded in 2 steps.

The assembly of a thicker grooved plate and a spacer to lock the machining spindle may pose an issue if its loading macro is not modified. The translation of the pinned axis in its bore also helps to compensate for machine misalignment during tool locking. The complete removal of this degree of freedom must, therefore, be combined with a gradual locking process as shown in Fig. 25. Tool locking and carrier removal are performed in two steps instead of one.

### 6.3 Wiring the spindle and the chip collection system

The machining spindle motor (EMAX CF2822) needs to be powered with 12 V 16A. It can be connected to a laboratory power supply or a cheaper 24 V-12 V step-down converter. The motor is controlled through its ESC with a PWM signal from the Duet board. Spindle control is carried out with the machine's tool change macros (turning on in the loading macro and turning off in the unloading macro). No setpoints are present in the G-code. Depending on the type of ESC, the spindle will respond within a precise PWM signal range, allowing for tool speed regulation. In the proposed setup, the spindle has a minimum speed of 1390 rpm at 55 PWM and reaches a maximum of 10858 rpm at 155 PWM. The printer's configuration file “config.g” will need to be modified to declare the new tool and initialize the spindle when the machine is turned on. The dust extraction system is connected to a 24 V/220 V relay, which is in turn controlled by a PIN on the machine's board. Like the spindle, turning the dust extraction on and off is managed with the loading and unloading macros.

An example configuration file and the loading and unloading macros including spindle and vacuum control are available on the Mendeley repository.

### 6.4 Tool offset adjustment

The use of a multi-tool printer inevitably requires adjusting the tools relative to each other in space. The most effective method for setting the tool origin for the spindle is to print a simple square part and, after checking the dimensions of the part, approach the running endmill to different faces of the part and note the XY position. The XY positions of the tools can then be corrected comparing those XY positions with the printing Gcode.

### 6.5 Additive process settings

In the context of hybridizing FFF and CNC processes, the printing nozzle must deposit more material than would have been done in simple additive manufacturing so that it can be machined afterward. The authors recommend an overthickness of 0.25 mm in radius.

### 6.6 Safety basics

The installation of a door on the ToolChanger frame is highly recommended to minimize risks during machining phases and safety glasses must be worn at all time. An error in tool height can lead to considerable damage to the machine's bed, and in case the endmill reaches it, it can result in electrical short circuits. Using a raft to compensate for potential errors in the Z-axis is advised. Careful adjustment of tool loading and unloading macros is necessary to prevent tool drops. A crash or spindle motor blockage can lead to a backlash in the controller or power supply being used.

## 7 Validation and characterization

For the characterization of the spindle's performance, test specimens are produced using 3 types of processes: regular FFF additive manufacturing, sequential hybrid and layer by layer hybrid. Since, currently, the only machining operations available in layer-by-layer hybridization (SuperSlicer) are contouring operations, only the faces affected by these operations will be analyzed (faces perpendicular to the build plate). All tests were conducted using Nanovia's PETG Carbon filament, and the printing parameters are summarized in the Table 1. The printing parameters remain unchanged between FFF and hybrid processes. Production times increases significantly depending on the process: 1h20min for simple FFF, 1h42min for sequential hybrid and 3h40min with layer by layer hybrid.

**Table 1:** Summary of the used manufacturing parameters.

Printing parameters	
Layer height	0,2mm
Extrusion T°	215°
Nozzle Ø	0,6 mm
Bed T°	80°
Printing speed	35 mm/s
Infill %	32 %
Machining parameters	
End mill Ø	3,17 mm
Number of effective teeths	1
Cutting speed Vc (m/min)	108 m/min
Spindle speed	10800 rpm
Feed rate	12 mm/s
Axial depth of cut	0,2 mm
Radial depth of cut	0,25 mm
Height of cut (mm)	0,2 (Layer by layer hybrid)/4/9/8 (Sequential Hybrid)

**Fig. 26:**
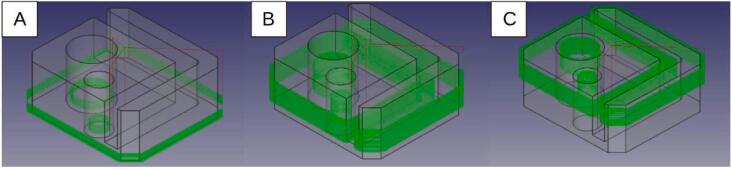
Distribution along the Z axis of machining phases programmed with FreeCAD, from left to right: A) machining from Z = 0.5 to 4.5 mm, B) machining from Z = 4 to 13.5 mm, C) machining from Z = 13 to 21 mm.

For the hybrid processes, the depth of cut is 0.2 mm. The machining is programmed using SuperSlicer for the layer-by-layer hybrid and FreeCAD for the sequential hybrid. For the latter, machining is carried out in three sequences integrated into the printing process at different heights: phase A from 0.5 to 4.5 mm (Fig. 26, A)/ phase B from 4 to 13.5 mm (Fig. 26, B)/ phase C from 13 to 21 mm (Fig. 26, C). All machining operations are performed in “climbing” mode.

**Fig. 27:**
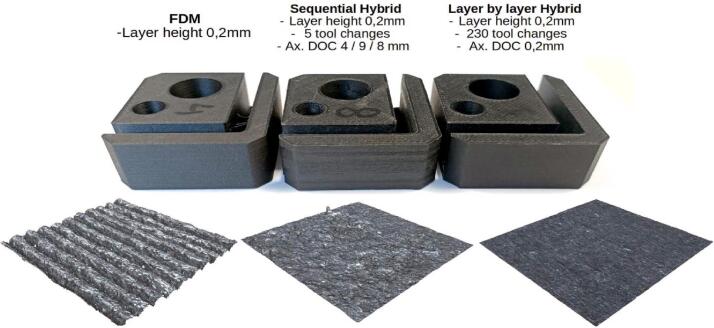
Different processes, from left to right: Simple FFF printing, Sequential hybrid, Layer-by-layer hybrid.

**Fig. 28:**
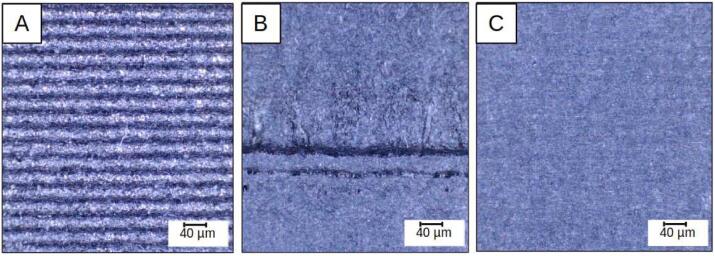
Surfaces perpendicular to the bed observed at x20 magnification: Simple FFF printing (A), Sequential hybrid (B), Layer-by-layer hybrid (C).

The samples produced, as well as the surface topologies, captured with an Alicona Portable RL optical profilometer, are presented in Fig. 27. Photographies taken with a USB digital microscope at a magnification of x20 are shown in Fig. 28.

**Fig. 29:**
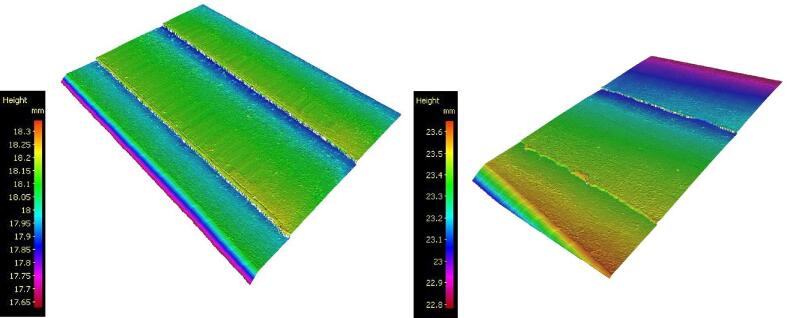
The topography of two opposite lateral faces on a sample produced using sequential hybridization, Alicona Portable LR.

The machined surfaces in the “sequential” hybridization show signs of burning (Fig. 28, B) on areas that have undergone repeated contact with the cutting tool. These are caused by the lack of lubrication and a low feed rate (12 mm/s) given the high rotational speed (10800 rpm). Unfortunately, this speed cannot be increased due to the machine's lack of rigidity and the resulting vibrations. Significant shape defects are also visible when two machining phases meet. The images in Fig. 29, taken with an optical profilometer on two opposite faces, highlight that these defects are due to the flexing of the entire carrier when machining passes occur at significant Z heights (4–9.5 mm). The machine's chassis, originally designed for FFF 3D printing, is not capable of supporting the cutting forces inherent in the sequential hybridization as programmed in these tests.

On the other hand, the capability of the layer-by-layer hybrid process (Fig. 28, C) to achieve high-quality surface finishes despite a significant number of tool changes (230 for this 21 mm high part) is confirmed. This is made possible by the repeatability of the ToolChanger's positioning and the reliability of the milling spindle proposed by the authors. Layer-by-layer hybridization also benefits from the fact that, by operating on thin layers, the cutting conditions are particularly favorable both thermally and in terms of the forces generated by material removal.

**Fig. 30:**
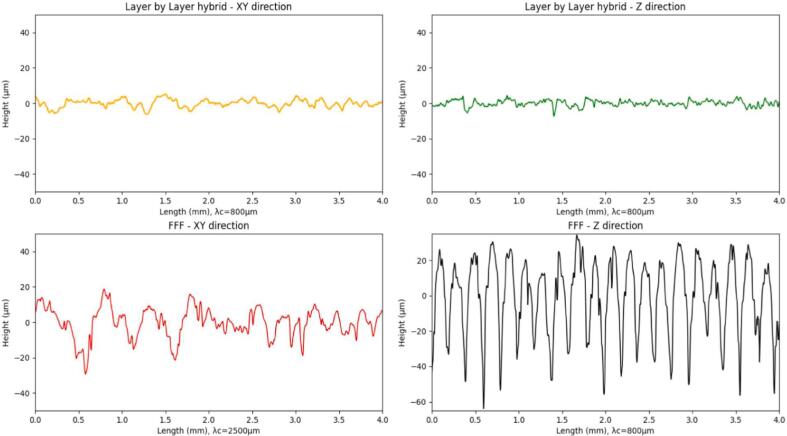
Ra values for the FFF and layer-by-layer hybrid processes.

Surface finish measurements were carried out using the Alicona Portable LR optical profilometer on the surfaces perpendicular to the build platform for both FFF and layer-by-layer hybrid processes. Due to the significant shape defects in the sequential hybridization, the produced sample is considered non-compliant and is not analyzed here. For both processes, three measurements were taken in the Z-axis (build direction) and in the XY-axis (deposition and/or machining direction). The average values of the roughness parameters are summarized in Table 2. The profiles with the highest Ra values are shown Fig. 30.

**Table 2:** Roughness parameters in µm for the layer-by-layer hybrid and FFF processes in the Z-axis (build) and X-Y-axis (deposition/machining) directions.

Layer by layer hybrid								
Parameters	Ra	Rt	Rp	Rv	Rz	Rsm	Rsk	Rku
Z direction	1,374	13,326	7,067	6,258	9,251	235,667	−0,07	4,24
XY direction	1,638	12,144	6,528	5,617	9,155	254,707	−0,14	3,28
								

FFF								

Parameters	Ra	Rt	Rp	Rv	Rz	Rsm	Rsk	Rku

Z direction	17,988	98,928	35,845	63,082	85,530	205,762	0,04	1,86
XY direction	6,869	51,880	22,665	29,215	41,574	356,091	−0,32	3,04

The measurement of surface roughness was performed according to ISO 4287 standards, and the definition of the assessed parameters are provided in Table 3. Layer thicknesses and axial pass depths of 0.2 mm for the faces in the Z construction direction generate periodic profiles, as confirmed by Rsm values of 235.66 µm and 205.75 µm (hybrid, FFF). A Gaussian filter λc = 800 µm with an evaluation length of 4 mm is applied for these measurements. In the context of measurements for the XY direction, filter choices are made through iterations. The use of λc = 800 µm and λc = 2500 µm with evaluation lengths of 4 and 12.5 mm (hybrid – FFF) allows obtaining Ra, Rz, and Rsm parameters within the specified ranges defined by the standards. In Fig. 30 the profile of the FFF surface in the XY direction have been cropped to match the scale of the others profiles and to facilitate readability.

**Table 3:** Roughness parameters assessed, based on “Surface Texture – Characterization, R1230, Techniques de l’ingénieur.” [24].

Parameter	Definition
Ra	Arithmetic Mean Deviation of the Evaluated Profile
Rt	Total Profile Height
Rp	Maximum Profile Peak Height
Rv	Maximum Profile Valley Depth
Rz	Maximum Profile Height
Rsm	Average Element Width of the Profile
Rsk	Asymmetry Factor of the Evaluated Profile
Rku	Flattening Factor of the Evaluated Profile

**Fig. 31:**
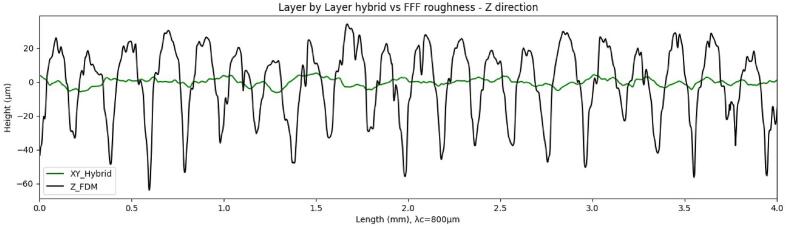
Roughness profiles of FFF vs. layer-by-layer hybrid in the Z-axis build direction.

Table 2 shows that hybridization tends to significantly improve the roughness parameters, with an improvement factor of 10 for most parameters. Comparing the Rt values for the XY and Z directions of each process illustrates the significant reduction in surface texture amplitude, which is also visible in Fig. 31 with the superposition of roughness profiles in the construction direction with and without hybridization. The data also show that with hybridization, parameters in the construction direction (Z) and in the deposition/machining direction (XY) tend to become uniform. The combination of FFF and CNC processes allows for the smoothing of vertically built faces (contouring operations only) and eliminates the typical FFF “stair-case effect”. The obtained Ra values (1.37 and 1.63 µm) are higher than values reported in the literature for works specifically aiming to optimize surface roughness in FFF, even for thinner layer thicknesses, smaller nozzle diameters, and measurements on faces parallel to the construction plane without the stair-case effect [24], [25], [26], [27], [28], [29]. The Ra values obtained with layer-by-layer hybridization fall within the typical range of values for traditional “conventional” or “climbing” machining processes [18].

**Fig. 32:**
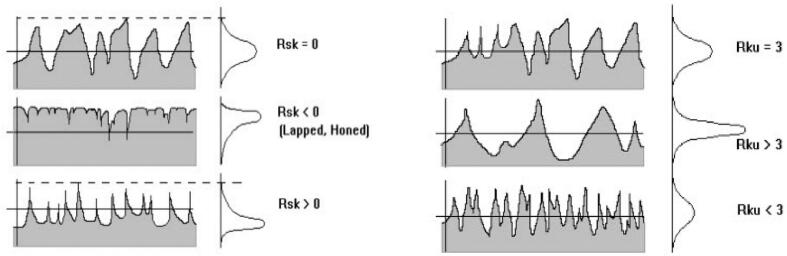
Rsk & Rku parameters illustration [22], [33].

In the Z-axis construction direction, the changes in the Rsk and Rku parameters (illustrated in Fig. 32), which respectively represent asymmetry and flatness factors of the profile [30], also indicate a shift in the profile morphology. The transition to Rsk < 0 and Rku > 3 suggests the presence of a “full” and “tight” profile, potentially affecting wear resistance and mechanical behavior in applications involving friction and lubrication for example [31], [32].

**Fig. 33:**
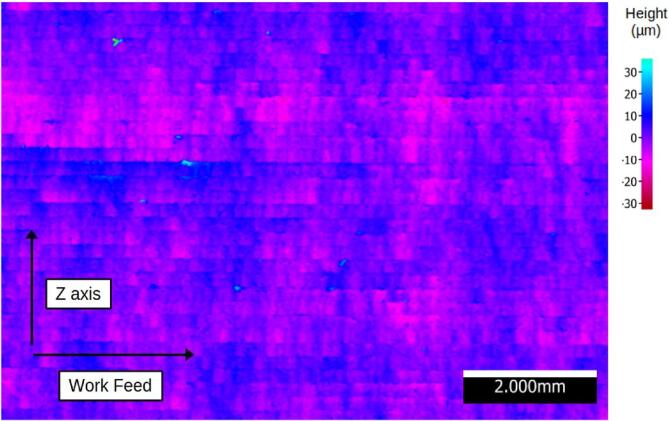
Topology of a surface machined in layer-by-layer hybrid, Alicona Portable LR.

A surface measurement taken on one of the faces produced in the layer-by-layer hybrid (Fig. 33) reveals that the shape defect identified for the sequential hybrid (Fig. 29) also occurs, to a lesser extent (±10 µm), for the layer-by-layer hybrid. This defect could be mitigated by optimizing the manufacturing parameters: Table 2, for example, the Rv of 63 µm in the Z direction for the FFF-produced part indicates that the initial radial pass depths set at 250 µm (Table 1) could be reduced. This would help reduce cutting forces and alleviate ripple defects.

## 8 Conclusions

The evaluation of the machining spindle's performance focused on the analysis of surface roughness to assess the contribution of machining to FFF 3D printing and the difference between two different types of hybrids.

• The results obtained show that the spindle presented in this work, which is more rigid and reliable than the one originally proposed by E3D, is capable of successfully carrying out a hybrid process that significantly improves the surface roughness of parts during contouring operations. The comparison made with the same cutting and feed rates between the “sequential” hybrid (machining occurring over several millimeters in height) and the layer-by-layer hybrid process shows that the latter is significantly advantaged by the fact that it operates on thin layers, creating particularly favorable cutting conditions both thermally and in terms of the forces generated by material removal. This is also a significant advantage, as the entire process is carried out on a machine with a low-rigidity frame.
• Layer-by-layer hybrid appears to be a suitable solution for an FFF/CNC hybrid technology because the reduction in temperature rise during machining means that there is no need for lubrication, which is a considerable advantage in the context of printing with low-melting-point polymers where the use of lubrication would cause inter-layer adhesion problems.

## CRediT authorship contribution statement

**Luis Vincent Tejada Martinez:** Writing – original draft, Validation, Methodology, Investigation, Data curation, Conceptualization. **Jean-François Witz:** Supervision. **Denis Najjar:** Writing – review & editing. **Xavier Boidin:** Writing – review & editing. **François Lesaffre:** Resources. **Vincent Martin:** Resources. **Sophie Badin:** Resources. **Emmanuel Berte:** Resources.

## Declaration of competing interest

The authors declare that they have no known competing financial interests or personal relationships that could have appeared to influence the work reported in this paper.

## Footnotes

1 https://www.eu.snapmaker.com, https://www.5axismaker.co.uk, https://www.zmorph3d.com, https://www.www.4dpioneers.com/copy-of-4shift-1, https://www.namma-france.com/.

2 https://e3d-online.com/blogs/news/asmbl.

3 https://www.thingiverse.com/thing:4206827.

4 https://wiki.freecadweb.org/Path_Workbench/en.
